# Preparation and Properties of High-Temperature-Resistant, Lightweight, Flexible Polyimide Foams with Different Diamine Structures

**DOI:** 10.3390/polym15122609

**Published:** 2023-06-08

**Authors:** Shuhuan Yun, Xianzhe Sheng, Shengli Wang, Xing Miao, Xuetao Shi, Yongsheng Zhao, Jianbin Qin, Guangcheng Zhang

**Affiliations:** School of Chemistry and Chemical Engineering, Northwestern Polytechnical University, Xi’an 710072, China

**Keywords:** polyimide foams, molecular structures, thermo-foaming, flame retardant, thermal insulation

## Abstract

Polyimide foam (PIF) is a rising star in high-end applications such as aerospace thermal insulation and military sound absorption. However, the basic rule on molecular backbone design and uniform pore formation of PIF still need to be explored. In this work, polyester ammonium salt (PEAS) precursor powders are synthesized between alcoholysis ester of 3, 3′, 4, 4′-benzophenone tetracarboxylic dianhydride (BTDE) and aromatic diamines with different chain flexibility and conformation symmetry. Then, a standard “stepwise heating” thermo-foaming approach is used to prepare PIF with comprehensive properties. A rational thermo-foaming program is designed based on in situ observation of pore formation during heating. The fabricated PIFs have uniform pore structure, and PIF_BTDA-PDA_ shows the smallest size (147 μm) and narrow distribution. Interestingly, PIF_BTDA-PDA_ also presents a balanced strain recovery rate (SR = 91%) and mechanical robustness (0.051 MPa at 25% strain) and its pore structure maintains regularity after 10 compression–recovery cycles, mainly due to high rigidity of the chains. Furthermore, all the PIFs possess lightweight feature (15–20 kg∙m^−3^), good heat resistance (*T*_g_ at 270–340 °C), thermal stability (*T*_5%_ at 480–530 °C), thermal insulation properties (λ = 0.046–0.053 W∙m^−1^K^−1^ at 20 °C, λ = 0.078–0.089 W∙m^−1^K^−1^ at 200 °C), and excellent flame retardancy (LOI > 40%). The reported monomer-mediated pore-structure control strategy can provide guidelines for the preparation of high-performance PIF and its industrial applications.

## 1. Introduction

Polymer foam is a porous material based on polymer resin with a large number of internal pores, which has received much attention because of its many excellent properties, such as low density, high specific strength, corrosion resistance, solvent resistance, and thermal insulation [1,2]. Traditional polymer foam includes polyvinyl chloride (PVC [3]) foam, polyethylene (PE [4]) foam, polystyrene (PS [5]) foam, polyurethane (PU [6]) foam, etc. Many polymer foam materials have been used in the field of housing construction for heat insulation, sound absorption, and noise reduction. With the development of the economy, society, science, and technology, people are increasingly demanding the safety, stability, and functionality of polymer foam materials, and traditional polymer foam no longer meets people’s requirements.

The modification of traditional polymer foams to improve their mechanical properties is an economical and effective method, which includes fiber-reinforced modification (glass-fiber-reinforced, nylon-fiber-reinforced), particle-reinforced modification (calcium carbonate ion reinforced, hollow glass microsphere reinforced, and nanoparticle-reinforced), and co-blending modification. The mechanical properties of the foams are significantly improved by the modified methods, but such methods do not change the molecular structure of the resin matrix; their heat resistance is not effectively improved, the phenomenon of foam softening occurs at high temperatures, and the foam structure changes drastically, which is no longer of practical significance. Several studies have shown that the compressive and shear properties of foam materials decrease significantly at high temperatures. For example, the compressive strength and modulus of PET foams are reduced to about 10% of the ambient temperature values at 100 °C [7]. Similarly, both strength and modulus in shear and compression present an approximately linear reduction with temperature in the 20–200 °C range [8].

Polyimides (PI) are a class of high-performance polymers that exhibit excellent mechanical, thermal, and chemical properties. Due to these outstanding characteristics, PI has found extensive applications in aerospace, electronics, advanced warships, rail transportation, and other high-tech fields. In recent years, there has been increasing interest and urgent need for lightweight and flexible high-temperature-resistant materials [9,10,11]. Polymer foams and aerogels have been developed to adapt to harsh working conditions [12,13,14,15]. For example, the new warplane engine casing needs foam that can withstand high temperatures above 300 °C to insulate and reduce noise. The ship’s cabin needs a flame-retardant insulation layer, a pipe protection layer, a radiation shield for the nuclear reactor, etc. Polyimide foam (PIF) shows great advantages in both superior properties and industrial compatible preparation. Apart from the imide rings in PI chain backbones, further foaming technology results in the pore structure with gas as the dispersed phase that endows PIFs with light weight, outstanding thermal insulation, flame retardancy, corrosion resistance, radiation resistance, chemical stability, and many other characteristics [9,16,17]. The pore structures, including open, closed and open/closed combined multiscale pore structures, in PIFs can extend their applications in many fields to realize weight reduction, high-temperature electronic substrates, thermal insulation, and sound absorption [18,19,20].

Recently, there are several methods that have been reported for the preparation of PIFs, including chemical foaming and physical foaming. The chemical foaming method involves mixing a PI precursor with a foaming agent which generates gas upon heating and result in the formation of cellular structure while physical foaming incorporates a blowing agent or supercritical fluid to produce highly uniform and interconnected foam structures. Typical approaches can be mainly divided into one-step method [21], two-step method [18], supercritical fluid foaming method [22], etc. Research on PIFs is still in an early stage, but there is growing interest in this area due to their potential in various applications. Xiang et al. [23] prepared isocyanate-based polyimide flexible foams with different isocyanate index by one-step method, achieve high open porosity and low density. However, isocyanates with high chemical activity are prone to participate in a variety of side reactions to form a large number of urea groups and carbamate groups in the system [24,25], leading to a significant reduction in the heat resistance and flame retardancy of PIFs.

The two-step method mainly refers to the powder/microsphere foaming method, which starts with the synthesis of the PEAS precursor powders from monomers such as aromatic dianhydride and diamine, followed by the preparation of PIFs in an oven by thermal foaming, microwave foaming, or a combination of both. Compared with the one-step isocyanate-based polyimide foam, the two-step polyimide foam has better thermal stability, heat resistance, flame retardancy, and mechanical properties [26].

Currently, plenty of studies have focused on finely tuning pore structures and further improving the mechanical properties and thermal stability of PIFs. Liu et al. [27] selected the rigid diamine monomer 2-(4-aminophenyl)-5-amino benzoxazole (DAPBO) to partially replace 4, 4′-diaminodiphenyl ether (ODA) in the BTDA-ODA system with different molar ratios and prepared a series of PIFs by the powder method and subsequent thermo-foaming. The results showed that a proper monomer ratio for DAPBO:ODA (3:2) is important to final pore structure and physical properties because of the balance between rigidity and flexibility of molecular chains to activate uniform foam bubbles. Ni et al. [28] prepared three PIFs with different backbone chains by microwave-assisted powder thermo-foaming method based on the reaction between 3, 3′, 4, 4′-benzophenone tetracarboxylic dianhydride (BTDA) with ODA, 4, 4′-diaminodiphenyl methane (MDA) and 4, 4′-diamino-2, 2′dimethylbiphenyl (DMBZ), respectively. It was shown that the flexibility of foam cells is strongly dependent on the chain rigidity that can be assessed by the glass transition temperature *T*_g_. Obviously, proper selection of dianhydride or diamine monomer leads to the difference in PIFs structure and performance, and the molecular structure is the fundamental reason affecting the structural and performance of PIFs. However, some of the diamine monomers used in the above studies have complex molecular structure and long synthesis steps, which makes it difficult to achieve large-scale production and industrialization of PIFs.

During the powder thermo-foaming process, the foaming temperature, heating rate, and holding time are the key factors to maximize the excellent performance of PIFs and depend on the particle size and distribution of precursor powders, residual solvent content, melt viscosity, etc. [19,29,30]. Therefore, industrial-scale monomer selection and corresponding thermo-foaming technology should be further explored.

In this study, commercially available and low-cost dianhydride and diamine monomers were selected for the fabrication of lightweight, high-temperature-resistant, thermally insulating, and flame-retardant PIFs by the two-step powder thermo-foaming method. The dianhydride was fixed as 3, 3′, 4, 4′-benzophenone tetracarboxylic dianhydride (BTDA) and chemically modified as alcoholysis ester (BTDE). Five selected diamine monomers with different chain flexibility and conformation symmetry can further react with BTDE to form polyester ammonium salts precursor (PEAS) powders. An in situ microfoaming process detected by optical microscope is conducted to design an optimized foaming process. Then, we discuss how the selected monomers influence pore structure and final mechanical performance of PIFs through detailed the molecular chain flexibility and foaming dynamics. In this way, a general thermo-foaming principle is further clarified and is of course helpful to finely tune multiscale pore structures of PIFs. Additionally, some other physical properties, including heat resistance, thermal stability, thermal insulating property, and flame retardancy, are compared among different PIFs. Based on analysis of the comprehensive properties, it is clear to see the potential in special working condition or industry and we inevitably provide guidelines for the industrial-scale production of high-performance PIFs. We also provide a solid theoretical and practical basis for future work on the more stable preparation and development of more heat-resistant polyimide foams.

## 2. Materials and Experimental

### 2.1. Materials

The 3, 3′, 4, 4′-benzophenone tetracarboxylic anhydride (BTDA) was purchased from Tianjin Zhongtai Material Technology Co., Ltd. (Tianjin, China) and dried at 100 °C for 12 h before use. The 4, 4′-diaminodiphenyl ether (ODA), 4, 4′-diaminodiphenyl methane (MDA), m-phenylenediamine (MPD), 4, 4′-diaminodiphenyl sulfone (DDS), and p-phenylenediamine (PDA) were purchased from Tianjin Zhongtai Material Technology Co., Ltd. (Tianjin, China). The ring-opening catalyst 2-methylimidazole (2-MI) was used as solvent and foaming agent and was purchased from Aladdin Reagent Co., Ltd. (Shanghai, China). Anhydrous ethanol (EtOH) was purchased from Guangdong Guanghua Technology Co., Ltd. (Guangdong, China). Silicone oil AK8805 was purchased from Xuzhou Yihuiyang New Material Co., Ltd. (Xuzhou, China).

### 2.2. Synthesis of Polyester Ammonium Precursor Powders and Preparation of Foams

Under the protection of nitrogen atmosphere, the appropriate amount of anhydrous ethanol was firstly added into a three-neck flask that was immersed in an oil bath at 70 °C. Then, BTDA (32.22 g, 0.1 mol) and ring-opening catalyst 2-MI (0.03 g) were added under continuous mechanical stirring and cool reflux for 2 h to obtain 3, 3′, 4, 4′-benzophenone tetracarboxylic acid diester (BTDE), until the solution gradually changed from white to light yellow. At this time, equimolar diamine ODA, MDA, MPD, DDS, or PDA was added for further stirring for 2 h, and then silicone oil AK8805 (0.5 mL) was added to as a surfactant for 20 min to obtain five different polyester ammonium salt (PEAS) precursor solutions. After the reaction was completed, the resulting PEAS powders were obtained through rotary evaporation. Before thermo-foaming, the PEAS powders were finally ground and sieved to obtain a fine powder size. Because of the potential hydrogen bonding interaction of the sulfone group in the DDS with ethanol, and the molecule is prone to forming hydrogen bonds with the hydroxyl group in ethanol, the PEAS_BDTA-DDS_ precursor powders were dried twice.

**Figure 1:**
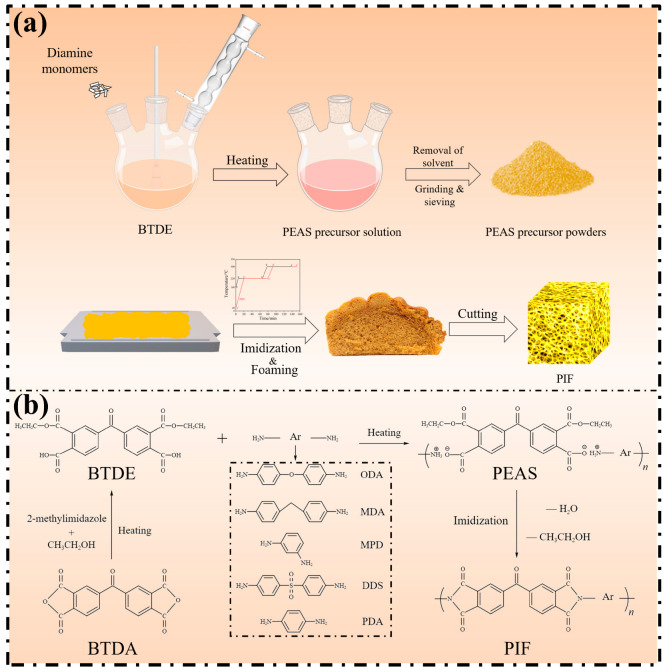
Synthesis routes and preparation process of PIFs with different diamine structures: (**a**) preparation process; (**b**) synthesis routes.

During the thermofoaming process, the PEAS powder was firstly spread on the graphite plate and together transferred into a temperature-controlled oven for simultaneous imidization and thermal foaming. The oven was programmed under a preset heating protocol, i.e., firstly to 160 °C in advance for preheating the powders, then the temperature rose to 220 °C and was kept constant 1 h, then, finally, the temperature further rose to 300 °C and was kept constant for another 1 h to complete the stepwise-heating thermo-foaming process. In addition, the forming process of PIF_BTDA-DDS_ was slightly adjusted and it was placed in the oven at room temperature to finish the preheating procedure. The relevant preparation process is shown in Figure 1.

### 2.3. Characterization

#### 2.3.1. Fourier-Transform Infrared Spectroscopy (FTIR)

The chemical structure of PEAS powders and PIFs was analyzed using an infrared spectrometer (Nicolet iS50 FTIR, Thermo Scientific, Massachusetts, USA) spectrometer under an attenuated total reflection (ATR) detection mode. The testing wavenumber ranges from 500 cm^−1^ to 4000 cm^−1^.

#### 2.3.2. Scanning Electron Microscopy (SEM)

A scanning electron microscope (VEGA3 LMH, TESCAN, Brno, Czech) was used to observe the particle morphology of the PEAS powders and the cellular structure of the different PIFs. Before observation, gold sputtering was finished on the surface of the powders and cryo-fractured surface of PIF samples. The pore size and size distribution were measured and counted by using ImagePro (Plus 6.0) software.

#### 2.3.3. Hot-Stage Optical Microscopy

The foaming behavior of the PEAS powders was in situ monitored using an optical microscope equipped with a hot-stage (Eclipse E400, Nikon, Tokyo, Japan). The temperature was gradually increased under a constant heating rate of 10 °C/min and the morphologies of the precursor powders at different temperatures were recorded using a color digital camera.

#### 2.3.4. Compression-Recovery Tests

The cyclic compression–recovery properties of PIFs were tested at room temperature using a universal testing machine (CMT 6303, Sans, Shenzhen, China). The PIF sample was tailored into a cube of 30 mm × 30 mm × 30 mm. The press head speed was set as 3 mm/min and compressed to a fixed maximum strain of 60% along the compression direction parallel to the cell growth direction. Each sample was compressed and released for 10 cycles. The test for each group of PIF samples was repeated at least five times. The apparent density of PIFs was calculated based on the mass/volume ratio, and strain recovery rates were calculated.

SR = *H*_1_/*H*_0_ × 100% (1)

In Equation (1), SR is the strain recovery rate, *H*_1_ is the height of foam after cyclic compression, and *H*_0_ is the height of foam before cyclic compression.

#### 2.3.5. Differential Scanning Calorimetry (DSC)

The simultaneous imidization and foaming process of the precursor PEAS powders was analyzed using a differential scanning calorimeter (Mettler DSC3, Mettler-Toledo, Zurich, Switzerland) under a simple heating run, and the glass transition temperatures of PIFs were also measured. All the tests were conducted under nitrogen atmosphere with a heating rate of 10 °C/min ranging from 25 °C to 350 °C.

#### 2.3.6. Thermogravimetric Analysis (TGA)

The weight loss was collected using a thermogravimetric analyzer (TG 209 F3, NETZSCH, Selb, Germany) under a dry nitrogen atmosphere with a flow rate of 20 mL/min. The samples were heated from 50 °C to 800 °C under a heating rate of 10 °C/min and the residual mass of each sample was recorded. Meanwhile, the precursor powders were tested by TGA to characterize the residual solvent content in the powder.

#### 2.3.7. Flame-Retardant Property

The flame-retardant properties of the foam samples were tested using a vertical combustion tester (ZR-02, Shanfang, Qingdao, China) and an oxygen index tester (ZR-01, Shanfang, Qingdao, China). The vertical burning test was performed by fixing the foam sample in size of 125 mm × 13 mm × 6 mm vertically at a distance of 10 mm above the methane flame with a flame action time of 25 s. The oxygen index measurement was performed according to GB/T 2406.2-2009 with a sample size of 80 mm × 10 mm × 10 mm.

#### 2.3.8. Thermal Insulation Property

The thermal conductivity of PIFs was, respectively, measured at 20 °C and 200 °C using a thermal conductivity tester in guarded hot plate method (DRH-100 and DRH-GW, Xiangke, Xiangtan, China). The tested sample size was 50 mm × 50 mm × 6 mm. To evaluate the thermal insulation of PIFs, the real-time temperature distribution of heated samples on a 200 °C hot plate was periodically recorded using a Testo 875-2i infrared camera (Ti480 PRO, Fluke, Everett, WA, USA).

#### 2.3.9. The Residual Solvent Content in the Powder

The precursor powder of mass (m_1_) was weighed and dried for 8 h at 90 °C. Then, the dried mass (m_2_) was weighed again, and the residual solvent content in the precursor powder was expressed as (m_1_ − m_2_)/m_1_ × 100%. Powder samples for each structure were measured three times.

## 3. Results and Discussion

### 3.1. Characteristic Absorption Peaks in FTIR Spectrum

**Figure 2:**
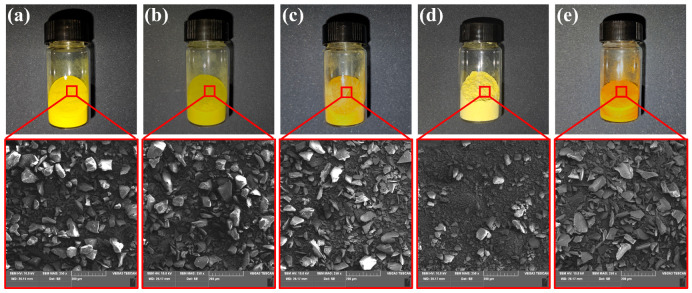
The digital photographs and SEM images of PEAS precursor powders: (**a**) PEAS_BTDA-ODA_; (**b**) PEAS_BTDA-MDA_; (**c**) PEAS_BTDA-MPD_; (**d**) PEAS_BTDA-DDS_; (**e**) PEAS_BTDA-PDA_.

In Figure 2, digital photographs of the appearance of the precursor powders and their microscopic morphology are shown. The prepared powders exhibit different colors due to the different diamine monomers; meanwhile, these precursor powders were ground and sieved by 150 mesh sieve, and it can be found that their particle sizes were all less than 100 μm, but also due to grinding and sieving, the morphology of the powders showed irregular blocks. The purpose of grinding and sieving is to obtain precursor powders with smaller particle size in order to increase their specific surface area and, thus, we expect to obtain a more uniform vesicular structure [31].

**Figure 3:**
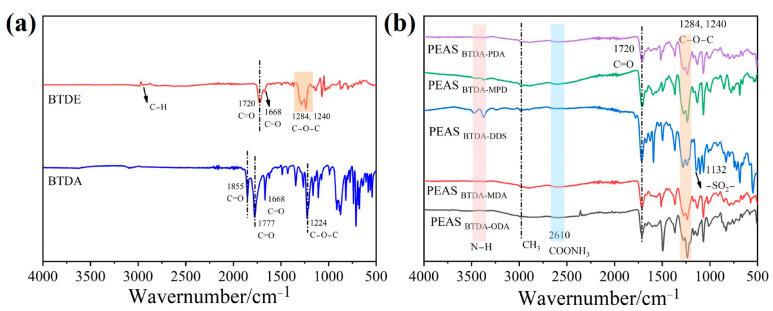
FTIR spectra of BTDA and BTDE (**a**) and PEAS precursor powders (**b**).

FTIR spectroscopy can provide important information about various functional groups. As shown in Figure 3a,b, the chemical structures of BTDA, BTDE, and PEAS with different structures were characterized using FTIR. Figure 3a shows that the characteristic peaks of C=O stretching vibrations of the five-membered cyclic anhydride in BTDA ν_as C=O_ and ν_s C=O_ are located at 1855 cm^−1^ and 1777 cm^−1^, respectively, and the characteristic peaks of C–O–C stretching vibrations are located at 1224 cm^−1^, and the peaks at 1668 cm^−1^ are the characteristic peaks of the ketone carbonyl group under the influence of benzene ring conjugation. The presence of the ester group is evidenced by the ν_C=O_ characteristic peak at 1720 cm^−1^ and the ν_as C-O-C_ characteristic peaks at 1284 cm^−1^ and 1240 cm^−1^ together, indicating that BTDA was alcoholically esterified and the five-membered ring of the anhydride in the molecule was opened to form BTDE. The subsequent appearance of the COONH_3_ groups with characteristic peaks near 2610 cm^−1^ and the N–H stretching vibrational absorption peaks located between 3300 and 3550 cm^−1^ indicate the successful synthesis of PEAS [32]. The molecular structure models and FTIR spectra of diamine monomers are shown in Figure S2.

### 3.2. Microfoaming Behavior of PEAS and the Optimized Thermo-Foaming Protocol

**Figure 4:**
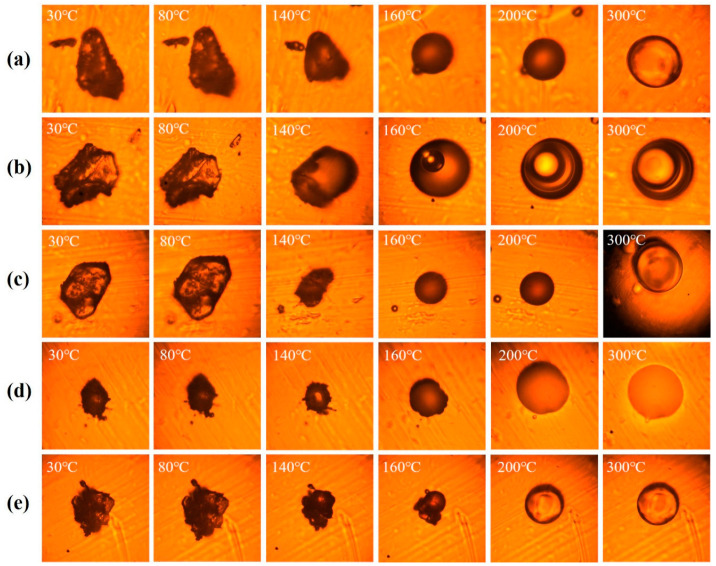
Microfoaming behavior of PEAS precursor powders: (**a**) PEAS_BTDA-ODA_; (**b**) PEAS_BTDA-MDA_; (**c**) PEAS_BTDA-MPD_; (**d**) PEAS_BTDA-DDS_; (**e**) PEAS_BTDA-PDA_.

To observe the foaming process of the precursor powders and the morphology of the melt and then to investigate the optimal foaming temperature for the preparation of PIFs with more uniform bubble morphology, the thermal foaming behavior of five different diamine structured precursor powders was traced using a visual study with a hot-stage polarizing microscope, and the morphological changes were recorded using a digital color camera, as shown in Figure 4.

From the micrographs, it can be seen that the foaming process of the precursor powders with different diamine structures is relatively similar and consistent with the foaming process of polymeric foams, which is roughly divided into three stages [33]: (1) Between 30–80 °C, as the temperature rises, the supersaturated gas dissolved in the resin matrix gradually transforms into the gas phase and migrates to the defects such as micropores or cracks inside the powders particles, forming bubble nuclei [34]; (2) when the temperature continues to rise to 200 °C in the process, the powders melt, melt viscosity decreases, the gas continues to precipitate from the resin matrix, converging to the bubble nucleus, bubbles overcome the liquid film interfacial tension, and external pressure continue to grow, and at the same time between the adjacent bubbles extrusion and merging, foam rapid expansion occurs [35]; (3) after 200 °C, the powders imide, and when the strength of the melt is not enough to support the stable foam it will break, and, finally, at 300 °C, the foam solidifies and the shape becomes stable.

**Figure 5:**
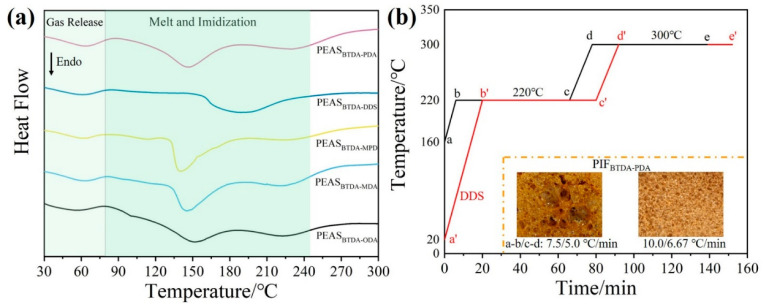
DSC curves of PEAS (**a**) and foaming temperature program set curves (**b**).

Meanwhile, the thermal changes of different structural precursor powders at different temperatures were analyzed by DSC. In Figure 5a, the precursor powders absorb heat at 30–80 °C, and the residual solvent in the powders is volatilized and released; the powders continue to absorb heat at 80–200 °C when melting occurs. In the case of the precursor powders, the powders continue to absorb heat and melt at 80–200 °C. After 200 °C, the powders undergo imide reaction, which is consistent with the microscopic thermal foaming behavior of the precursor powders (Figure 4). Combining the results of the visual study of the hot-stage polarization microscopy with the DSC analysis of PEAS precursor powders, the “stepwise heating” thermo-foaming process of different structured precursor powders was designed, as shown in Figure 5b. First of all, the oven should be preheated to 160 °C for preheating the powders, and then the spread powders should be quickly placed into the oven, then the oven will start to heat up to 220 °C according to the heating rate of section b–c, and is kept constant for 1 h. After the end of heat preservation, the oven will continue to heat up to 300 °C according to the heating rate of section d–e, and is also kept constant for another 1 h to complete the post-curing of the foam, and finally cools naturally to room temperature to obtain PIFs. In addition, the residual solvent content in the precursor powder also has an important effect on the thermo-foaming process. The residual solvent content in the precursor powder was measured, and the residual solvent contents of PEAS_BTDA-ODA_, PEAS_BTDA-MDA_, PEAS_BTDA-MPD_, PEAS_BTDA-DDS_, and PEAS_BTDA-PDA_ were 12.65%, 13.65%, 13.38%, 17.62%, and 12.95% (Table S1 and Figure S3), respectively. Among them, PEAS_BTDA-DDS_ had the highest residual solvent content due to the potential hydrogen bonding interaction of the sulfone group in the DDS with ethanol. Therefore, the PEAS_BTDA-DDS_ precursor powder needs to be warmed up simultaneously with the oven to reduce the residual solvent content in order to obtain homogeneous PIFs, while the rest of the structure will have too low residual solvent content if it is foamed by warming up, and finally the precursor powder cannot be foamed.

In the actual foaming process, it was found that the formation of the pore structure morphology is very sensitive to the change of the heating rate of the a–b and c–d sections, but the specific effect of the heating rate on the foaming process was not carried out in depth in this study, and this part of the study will continue in the subsequent work. The results of the foaming tests to determine the temperature rise rate of different structured PEAS precursor powder foaming processes are shown in Table 1.

### 3.3. Imidization, Foaming, and Cellular Structure of PIFs

**Figure 6:**
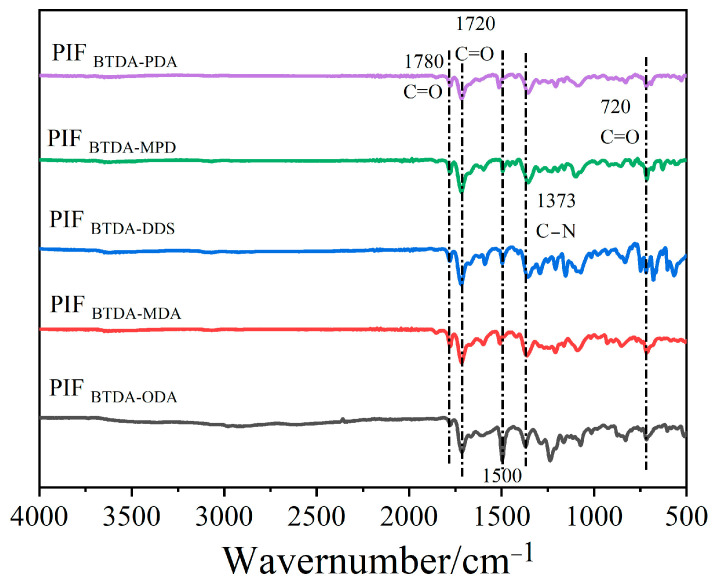
The ATR-FTIR spectra of PIFs with different diamine structures.

The chemical structures of different PIFs with diamine structures were characterized using ATR-FTIR. Figure 6 shows that the characteristic absorption peaks of the benzene rings are located at 1500 cm^−1^ and there is a significant increase in the characteristic peak at 1780 cm^−1^ compared to Figure 3b, indicating the imidization of the precursor powders during the high-temperature foaming process. Furthermore, asymmetric, symmetric stretching vibration absorption peaks and bending vibration peaks of C=O on the imide ring at 1780 cm^−1^, 1720 cm^−1^, and 720 cm^−1^ and stretching vibration absorption peak of C–N on the imide ring (1373 cm^−1^) were observed in Figure 6, indicating the successful imidization of PI [29]. The above findings indicate the successful imidization of PIFs with different diamine structures.

**Figure 7:**
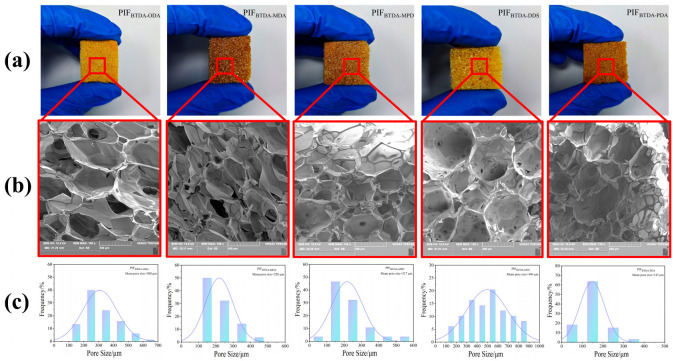
The appearance photos (**a**), SEM images (**b**), and pore size distribution (**c**) of different PIFs.

In Figure 7, the appearance photos and SEM images of PIFs with different diamine structures are presented, and the pore size distribution of the foams is counted. The results show that the PIFs with different diamine structures are yellow in color, exhibit low apparent densities (15–20 kg·m^−3^), and have micron-sized three-dimensional cellular pore structures, showing a combination of open and closed cellular structure. The average pore sizes of PIF_BTDA-ODA_, PIF_BTDA-MDA_, PIF_BTDA-MPD_, PIF_BTDA-DDS_, and PIF_BTDA-PDA_ were 308 μm, 220 μm, 217 μm, 496 μm, and 147 μm, respectively. It can be seen that, except for PIF_BTDA-DDS_, the average pore size of PIFs gradually decreased as the structural rigidity of the diamine monomer increased, which was caused by the difference in the structural rigidity of the molecular backbone chains between different structures; the stronger the rigidity of the backbone chains, the weaker the movement of the melt [19,28], which in turn affects the structure and morphology of the bubble pores. In addition to the molecular chains structure itself, another factor that affects the flowability of the melt is the residual solvent content in the precursor powders, which directly affects the flowability of the melt during the melting process. The reason why PIF_BTDA-DDS_ has higher molecular backbone chains rigidity but shows the largest average pore size is because of the high residual solvent content of PEAS_BTDA-DDS_, which make it difficult to extract solvents due to hydrogen bonding.

### 3.4. Pore Structure Regularity and Compression–Recovery Properties of PIFs

**Figure 8:**
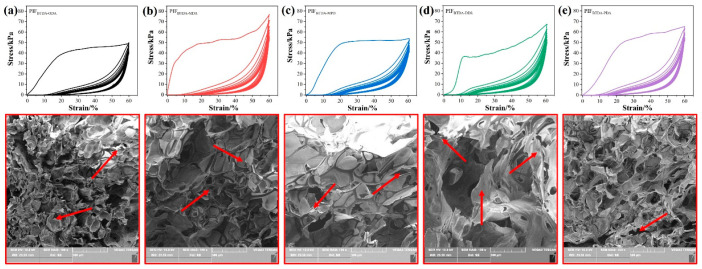
Cyclic compressive stress–strain curves of PIFs at 60% strain and SEM images of compressed PIFs: (**a**) PIF_BTDA-ODA_; (**b**) PIF_BTDA-MDA_; (**c**) PIF_BTDA-MPD_; (**d**) PIF_BTDA-DDS_; (**e**) PIF_BTDA-PDA_.

As shown in Figure 8, the elasticity and compression strength of PIFs with different structures were tested by performing cyclic compression–recovery tests at room temperature. PIFs with different structures were compressed and released for ten cycles continuously at fixed maximum strain of 60%, and the cyclic compression stress–strain curve was obtained. It can be seen that all PIFs have significant differences between the first compression process and the rest of the compression processes, which is due to the fact that the foam is a combination of the open and closed cell (Figure 7). The linear elastic behavior of the initial foam appears in the small strain region after compression, which is caused by the synergistic effect of the axial deformation of the pore edges [8], the pressure of the gas between pores in the open cell structure, the bending of the bubble wall, and the stretching of the bubble wall caused by the pressure of the gas inside the bubble in the closed pore structure. As the compression process continues into the plastic section, the bubble walls of the obtuse structure bend beyond the limit and crack, leading to the collapse of the bubble. The gas in the hole is compressed, creating a restoring force that makes the stress–strain curve less smooth. This is also an important reason why the deformation does not return to the initial state after the compressive force is released. According to the stress–strain curves, the 25% compressive strength and the residual strain after the first compression of different PIFs were calculated as PIF_BTDA-ODA_ (41.67 kPa, 5.71%), PIF_BTDA-MDA_ (49.90 kPa, 6.91%), PIF_BTDA-MPD_ (50.63 kPa, 6.74%), PIF_BTDA-DDS_ (38.67 kPa, 8.14%), and PIF_BTDA-PDA_ (51.17 kPa, 6.18%). The density and mechanical test results of the foam were summarized in Table 2. When the closed cell structure is destroyed, the open cell structure becomes the dominant structure of the PIFs. Under compressive load, the gas between the holes is squeezed and the pore edges are compressed and deformed. Under viscous flow hysteresis of fluid and polymer-deformation hysteresis, the foam undergoes elastic deformation and gradually recovers the foam deformation after the external force vanishes. However, plastic collapse occurs in a bubble hole when the moment generated by the compressive load is larger than the plastic moment at the hole edge [36].

As a result, the residual strain gradually increases with the increase in the number of cycles and eventually tends to stabilize. At the same time, the SR of PIF_BTDA-ODA_, PIF_BTDA-MDA_, PIF_BTDA-MPD_, PIF_BTDA-DDS_, and PIF_BTDA-PDA_ after ten cycles of compression and release were calculated as 87%, 89%, 90%, 85%, and 91%, respectively. At the same time, compared with Figure 7b, it can be obviously seen in Figure 8 that the bubble was damaged; in particular, the PIF_BTDA-DDS_ bubble with larger pore diameter showed the most serious bubble damage, while PIF_BTDA-PDA_ still showed a relatively complete bubble structure, indicating that its bubble structure had better stability. PIF_BTDA-PDA_ has a smaller residual strain and the highest compressive strength of 25%, which is attributed to the largest molecular backbone rigidity, smaller mean pore size, and narrow pore size distribution.

### 3.5. Molecular Origin of Pore Structure and Mechanical Performance of PIFs

**Figure 9:**
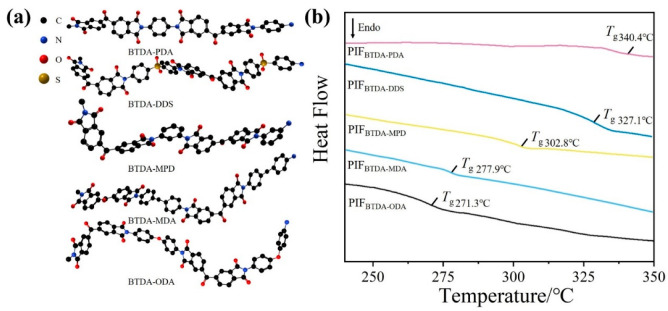
The conformational models of the main chains segment (**a**) and DSC curves (**b**) of PIFs with different diamine structures.

The glass transition temperatures (*T*_g_) of PIFs with different diamine structures were also measured by DSC, which was used to evaluate the rigidity of the molecular backbone chains. In Figure 9a, the conformational models of the main chains segment of PIFs with different diamine structures are shown. Figure 9b shows that the *T*_g_ of PIF_BTDA-ODA_, PIF_BTDA-MDA_, PIF_BTDA-MPD_, PIF_BTDA-DDS_, and PIF_BTDA-PDA_ were measured as 271.3 °C, 277.9 °C, 302.8 °C, 327.1 °C, and 340.4 °C, respectively. The results indicate that the prepared PIFs have different chain stiffness due to the chain flexibility of the stiffness of the selected diamines in the order of PDA > DDS > MPD > MDA > ODA. Herein, the increased *T*_g_ of PIFs indicates that the chain stiffness is effectively increased, thus endowing high mechanical strength and good pore structure regularity of PIF_BTDA-PDA_.

### 3.6. Thermal Stability and Thermal Insulative Performance of PIFs

**Figure 10:**
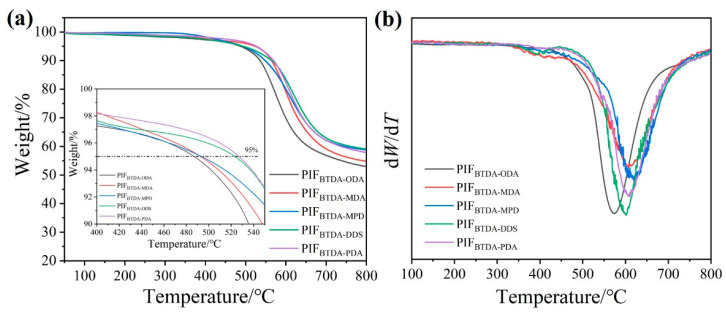
TGA (**a**) and DTG (**b**) curves of PIFs with different diamine structures.

TGA was used to characterize the thermal stability of PIFs, as shown in Figure 10. The results show that 5% mass loss (*T*_5%_) occurs between 480 and 530 °C, indicating that all PIFs have high thermal stability. The *T*_5%_ values of the PIFs with different chain structures were in the order of PIF_BTDA-PDA_ (526.9 °C) > PIF_BTDA-DDS_ (523.3 °C) > PIF_BTDA-MPD_ (493.3 °C) > PIF_BTDA-MDA_ (492.7 °C) > PIF_BTDA-ODA_ (487.1 °C). Similarly, it can be seen in the DTG diagram that the *T*_max_ of all the five PIFs is above 570 °C and the five diamine *T*_max_ is the temperature at which the thermal weight loss rate of the material is the fastest. Among the investigated samples, PIF_BTDA-PDA_ exhibits the highest *T*_5%_ and *T*_10%_ values, indicating that it has the best thermal stability.

**Figure 11:**
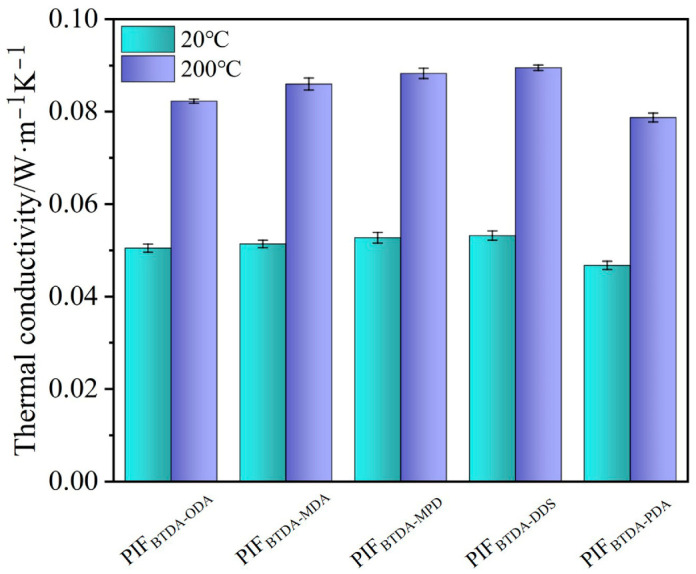
Thermal conductivity of PIFs at 20 °C and 200 °C.

Thermal conductivity is one of the key characteristics to assess the thermal insulating property of a material. In Figure 11, the thermal conductivity of PIFs was tested at 20 °C and 200 °C using room temperature and high-temperature thermal conductivity testers, respectively. The room-temperature thermal conductivity of all PIFs is around 0.05 W·m^−1^∙K^−1^, which is low enough and shows good thermal insulation and adiabatic properties. At 200 °C, the thermal conductivity slightly increases and is around 0.08 W·m^−1^∙K^−1^, indicating that the PIFs can still retain good thermal insulation properties at high temperatures. In addition, it was also found that the diamine rigidity is generally favorable for enhancing the thermal conductivity of PIFs, but the thermal conductivity of PIF_BTDA-PDA_ decreases since PIF_BTDA-PDA_ has the smallest average pore size and the narrowest pore size distribution. Thus, the reduction of thermal conductivity of PIF_BTDA-PDA_ may be ascribed to regularity of pore structure and higher gas density to restrain heat transfer [37].

**Figure 12:**
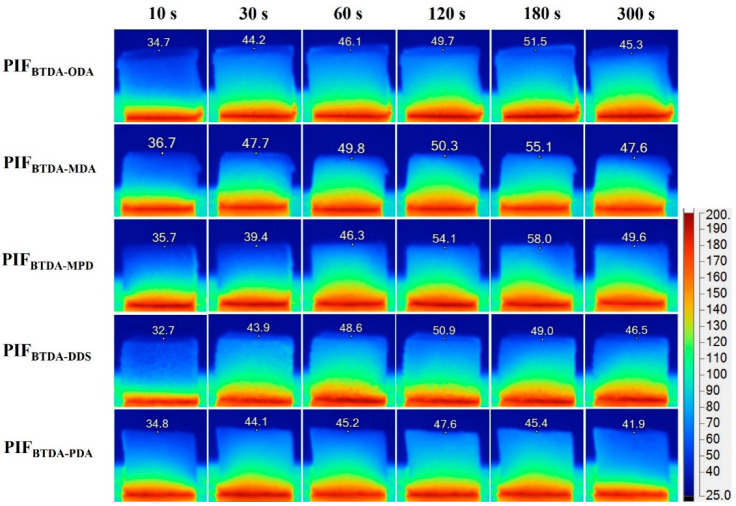
Infrared thermal image of the side of PIFs placed on a 200 °C hot table for 5 min.

In addition, the PIFs were placed on a hot plate at 200 °C and the thermal insulation property was checked using an infrared camera for real-time imaging (Figure 12). The change of foam temperature was detected from the side view of the foam at regular time intervals, and it was found that the upper surface temperature of the foam was in a low temperature range of 30–60 °C after long-time heat diffusion. After heating for 300 s, the upper surface temperature of PIF_BTDA-ODA_, PIF_BTDA-MDA_, PIF_BTDA-MPD_, PIF_BTDA-DDS_, and PIF_BTDA-PDA_ increased by 10.6 °C, 10.9 °C, 13.9 °C, 13.8 °C, and 7.1 °C respectively. All PIFs exhibit excellent thermal insulation performance while PIF_BTDA-PDA_ shows the best thermal insulation performance, which is consistent with the tested thermal conductivity. The above studies show that these lightweight and flexible PIFs can be widely used for thermal insulation in aerospace, rail transportation, etc.

### 3.7. Flame-Retardant Performance

The flame-retardant properties of the foam samples were determined using the vertical combustion method. No drops were seen during combustion, no smoke was observed with the naked eye, and the burning length of the samples was short (0.015 m), significantly less than the minimum 0.152 m required [38], indicating that the foam samples had good flame retardancy (the flame-retardant effect is shown in the Figures S4 and S5). The limiting oxygen index of the foam samples were tested with an oxygen index tester and the results are summarized in Table 3 together with the thermal performance data of the foams. The LOI of all the foam samples was above 40%, which also indicates that the foam samples have good flame-retardant properties.

As shown in Table 4, the prepared PIFs show better performance in terms of heat resistance, thermal stability, flame retardancy, and mechanical properties compared to those reported in the existing literature. Compared with PIF_BTDA-MDA_ in the literature, PIF with the same molecular structure in this study has higher *T*_g_ value and lower thermal conductivity at 200 °C, indicating better heat resistance and the ability to isolate heat. In addition, the fabricated PIFs are significantly superior to the isocyanate-based PIF regarding flame retardancy, thermal resistance, and thermal stability. Among them, the *T*_g_ of PIF_BTDA-PDA_ reached 340 °C, the thermal loss of weight temperature exceeded 520 °C, and the LOI > 42%. It was found that the unmodified PIF_BTDA-PDA_ still has comprehensive properties, including heat resistance, thermal stability, and cost control, compared to the modified PIF_BTDA-ODA/graphene_. Therefore, the PIFs prepared in this study can be produced at a large scale toward lightweight, heat resistant, thermal insulating, and flame-retardant materials with finely-tuned pore structure and good mechanical properties.

## 4. Conclusions

In summary, we firstly prepared precursor powders with different diamine structures using polyester ammonium salt (PEAS), then determined the appropriate foaming conditions by analyzing the thermal variation and microfoaming process of the precursor powders, and, finally, prepared PIFs with different molecular chain structures by the stepwise-heating thermo-foaming method. It was shown that the molecular chain structure along with residual solvent content and optimized foaming program are key factors to determine the structure and property of PIFs. The more uniform pore size and the narrower pore size distribution is of great importance for the mechanical performance of PIFs. The prepared PIFs also have excellent heat resistance and thermal stability. At the same time, PIFs have low thermal conductivity and excellent adiabatic and flame-retardant properties. This study presents a simple method to prepare lightweight, thermally insulative, and flame-retardant PIFs with good mechanical properties, providing a feasible solution for the demand of high-performance materials in extreme working conditions. However, the effect of the heating rate on the powder foaming and foam forming during the preparation of PIF remains unclear, while the process and design of foaming in the mold still need to be explored and studied, which will be the research direction of future work.

## Figures and Tables

**Table 1:** Residual solvent content and foaming heating rate of PEAS precursor powders with different structures.

Powders Samples	Residual Solvent Content/%	Heating Rate of a-b/°C·min^−1^	Heating Rate of c-d/°C·min^−1^
PEAS_BTDA-ODA_	12.65	7.5	5.0
PEAS_BTDA-MDA_	13.65	7.5	5.0
PEAS_BTDA-MPD_	13.38	10.0	5.0
PEAS_BTDA-DDS_	17.62	10.0	6.67
PEAS_BTDA-PDA_	12.95	10.0	6.67

**Table 2:** Density, 25% compressive strength, residual strain after the first compression, and SR of PIFs with different diamine structures.

Foam Samples	Density/kg∙m^−3^	Compressive Strength at 25%/kPa	Residual Strain after the First Compression/%	SR/%
PIF_BTDA-ODA_	15.60	41.47	5.71	87
PIF_BTDA-MDA_	17.11	49.90	6.91	89
PIF_BTDA-MPD_	16.45	50.63	6.74	90
PIF_BTDA-DDS_	19.96	38.67	8.14	85
PIF_BTDA-PDA_	16.26	51.17	6.18	91

**Table 3:** Thermal properties and limiting oxygen index (LOI) of PIFs synthesized from different diamines.

Foam Samples	*T*_g_/°C	*T*_5%_/°C	*T*_10%_/°C	*T*_max_/°C	R_800_/%-Nitrogen	LOI/%
PIF_BTDA-ODA_	271.3	487.1	535.7	573.5	53.0	41.8
PIF_BTDA-MDA_	277.9	492.7	548.1	607.3	58.8	40.1
PIF_BTDA-MPD_	302.8	493.3	564.3	615.3	59.1	42.5
PIF_BTDA-DDS_	327.1	523.3	564.4	602.3	54.9	42.6
PIF_BTDA-PDA_	340.4	526.9	569.3	609.6	57.9	42.8

**Table 4:** Various properties of polyimide foams.

Foam Samples	Density/kg∙m^−3^	Compressive Strength/kPa	*T*_g_/°C	*T*_5%_/°C	LOI/%	Thermal Conductivity/W·m^−1^∙K^−1^ at RT	Thermal Conductivity/W·m^−1^∙K^−1^ at 200 °C	Ref.
PIF_BTDA-ODA_	15.60	41.47 (25%)	271.3	487.1	41.8	0.0505	0.0823	This work
PIF_BTDA-MDA_	17.11	49.90 (25%)	277.9	492.7	40.1	0.0514	0.0859	This work
PIF_BTDA-MPD_	16.45	50.63 (25%)	302.8	493.3	42.5	0.0527	0.0883	This work
PIF_BTDA-DDS_	19.96	38.67 (25%)	327.1	523.3	42.6	0.0532	0.0895	This work
PIF_BTDA-PDA_	16.26	51.17 (25%)	340.4	526.9	42.8	0.0467	0.0787	This work
PIF_BTDA-MDA_	-	25.00 (10%)	255.9	493.0	-	0.0496	0.134	[39]
PIF_BTDA-ODA/graphene_	75	20.00 (10%)	260.0	480.0	-	-	-	[40]
PIF_(Isocyanate index 0.89–1.18)_	5.7–8.7	7.5–11.4 (20%)	-	288.5–320.0	25.3–28.3	-	-	[23]
Isocyanate-based PIF_ODA/PMDA_	13.7–16.49	33.0–45.7 (15%)	-	270.11–273.18	-	0.051–0.055	-	[41]
Isocyanate-based PIF at graphite (0–3.25%)	7.8–11.88	11.0–25.0	-	406.9–452.1 (*T*_10%_)	31.0–34.8	-	-	[42]

## Data Availability

The data are available from the corresponding author upon reasonable request.

## Supplementary Materials

The following are available online at https://www.mdpi.com/article/10.3390/polym15122609/s1, Figure S1: Appearance photos of PIF_BTDA-ODA_; Figure S2: Diamine molecular structure models (a) and FTIR spectrum of diamine monomers (b); Figure S3: The residual solvent contents of PEAS precursors; Figure S4: Flame retardant effect of PIF. Heating the foam with an alcohol lamp (a), the state of the heated surface after three minutes of heating (b), the state of the back of the foam after three minutes of heating (c); Figure S5: The state of the sample before vertical combustion (a), the state of the sample in vertical combustion (b), The state of the sample after 25 s vertical combustion (c); Table S1: Mass changes before and after drying and residual solvent contents of PEAS precursors.

## References

[1] Tomin M., Kmetty Á. (2021). Polymer foams as advanced energy absorbing materials for sports applications—A review. J. Appl. Polym. Sci..

[2] Ates M., Karadag S., Eker A.A., Eker B. (2022). Polyurethane foam materials and their industrial applications. Polym. Int..

[3] Shi A., Zhang G., Zhao C. (2012). Study of rigid cross-linked PVC foams with heat resistance. Molecules.

[4] McGee S.D., Batt G.S., Gibert J.M., Darby D.O. (2017). Predicting the Effect of Temperature on the Shock Absorption Properties of Polyethylene Foam. Packag. Technol. Sci..

[5] Wang G., Li W., Bai S., Wang Q. (2019). Synergistic Effects of Flame Retardants on the Flammability and Foamability of PS Foams Prepared by Supercritical Carbon Dioxide Foaming. ACS Omega.

[6] Xu Z., Chu F., Jiang S., Hu Y., Song L., Hu W. (2022). Mosquito’s eyes inspired, hydrophobic and multifunctional coating on flexible polyurethane (PU) foam: Highly efficient oil spills remediation and exceptional flame-retardant performance. Mater. Today Chem..

[7] Mazzuca P., Firmo J.P., Correia J.R., Garrido M. (2021). Mechanical behaviour in shear and compression of polyurethane foam at elevated temperature. J. Sandw. Struct. Mater..

[8] Mazzuca P., Firmo J.P., Correia J.R., Castilho E. (2021). Mechanical behaviour in shear and compression at elevated temperature of polyethylene terephthalate (PET) foam. J. Build. Eng..

[9] Li J., Zhang G., Li J., Zhou L., Jing Z., Ma Z. (2017). Preparation and properties of polyimide/chopped carbon fiber composite foams. Polym. Adv. Technol..

[10] Wang L., Hu A., Fan L., Yang S. (2013). Approach to produce rigid closed-cell polyimide foams. High Perform. Polym..

[11] Zhang H., Fan X., Chen W., Wang Y., Liu C., Cui B., Li G., Song J., Zhao D., Wang D. (2022). A simple and green strategy for preparing flexible thermoplastic polyimide foams with exceptional mechanical, thermal-insulating properties, and temperature resistance for high-temperature lightweight composite sandwich structures. Compos. Part B Eng..

[12] Mougel C., Garnier T., Cassagnau P., Sintes-Zydowicz N. (2019). Phenolic foams: A review of mechanical properties, fire resistance and new trends in phenol substitution. Polymer.

[13] Cafiero L., Iannace S., Sorrentino L. (2016). Microcellular foams from high performance miscible blends based on PEEK and PEI. Eur. Polym. J..

[14] Rai N., Chauhan I. (2023). Multifunctional Aerogels: A comprehensive review on types, synthesis and applications of aerogels. J. Sol Gel Sci. Technol..

[15] Shi B., Ma B., Wang C., He H., Qu L., Xu B., Chen Y. (2021). Fabrication and applications of polyimide nano-aerogels. Compos. Part A Appl. Sci. Manuf..

[16] Qiu G., Ma W., Wu L. (2020). Low dielectric constant polyimide mixtures fabricated by polyimide matrix and polyimide microsphere fillers. Polym. Int..

[17] Wang Y., Lu Y., Zhang J., Liang Y., Chi H., Xiao G. (2021). Enhanced toughness and gas permeabilities of polyimide composites derived from polyimide matrix and flower-like polyimide microparticles. Polym. Compos..

[18] Gu W., Wang G., Zhou M., Zhang T., Ji G. (2020). Polyimide-Based Foams: Fabrication and Multifunctional Applications. ACS Appl. Mater Interfaces.

[19] Ni L., Luo Y., Peng X., Zhou S., Zou H., Liang M. (2021). Investigation of the properties and structure of semi-rigid closed-cellular polyimide foams with different diamine structures. Polymer.

[20] Li J., Lin J., Wang Y., Chu W., Liu F., Wang B. (2022). Cross-Linked and Rigid Polyimide Composite Foams with Prominent Fire Resistant, Thermal Insulating, and Wave-Transparent Properties. ACS Appl. Polym. Mater..

[21] Liu H., Tian H., Yao Y., Xiang A., Qi H., Wu Q., Rajulu A.V. (2020). Polyimide foams with outstanding flame resistance and mechanical properties by the incorporation of noncovalent bond modified graphene oxide. New J. Chem..

[22] Teo N., Gu Z., Jana S.C. (2018). Polyimide-based aerogel foams, via emulsion-templating. Polymer.

[23] Xiang A., Li Y., Fu L., Chen Y., Tian H., Rajulu A.V. (2017). Thermal degradation and flame retardant properties of isocyanate-based flexible polyimide foams with different isocyanate indices. Thermochim. Acta.

[24] Sun G., Wang W., Zhang C., Liu L., Wei H., Han S. (2017). Fabrication of isocyanate-based polyimide foam by a postgrafting method. J. Appl. Polym. Sci..

[25] Liu X., Zhou P., Zhang R. (2022). Organic–Inorganic Hybrid Isocyanate-Based Polyimide Foams with Excellent Fire Resistance and Thermal Insulation Performance. ACS Appl. Polym. Mater..

[26] Luo Y., Ni L., Zhang X., Jiang X., Zou H., Zhou S., Liang M., Liu P. (2022). Fabrication of Rigid Polyimide Foams with Superior Compressive Properties. Ind. Eng. Chem. Res..

[27] Liu J.N., Wu D.Y., Liang W.H., Cao J.H. (2020). Effect of DAPBO segment on the structure and performance enhancement of powder foamed BTDA-ODA polyimide. J. Appl. Polym. Sci..

[28] Ni L., Luo Y., Qiu B., Yan L., Zou H., Zhou S., Liang M., Liu P. (2022). Combining Microwave-Assisted Foaming and Post Curing Process to Prepare Lightweight Flexible Polyimide Foams for Thermal Insulation Applications. Macromol. Mater. Eng..

[29] Ni L., Luo Y., Qiu C., Shen L., Zou H., Liang M., Liu P., Zhou S. (2022). Mechanically flexible polyimide foams with different chain structures for high temperature thermal insulation purposes. Mater. Today Phys..

[30] Zhan F., Youssef M., Shah B.R., Li J., Li B. (2022). Overview of foam system: Natural material-based foam, stabilization, characterization, and applications. Food Hydrocoll..

[31] Zhang Y.Z., Zhang G.C., He Z., Sun X.F., Rajaratnam M. (2010). Visualization Study of Solid Poly (Ester-Amine Salt) *Precursor Foam*. Process. Cell. Polym..

[32] Li J., Zhang G., Yao Y., Jing Z., Zhou L., Ma Z. (2016). Synthesis and properties of polyimide foams containing benzimidazole units. RSC Adv..

[33] Taki K. (2008). Experimental and numerical studies on the effects of pressure release rate on number density of bubbles and bubble growth in a polymeric foaming process. Chem. Eng. Sci..

[34] Colton J.S., Suh N.P. (1987). The nucleation of microcellular thermoplastic foam with additives: Part I: Theoretical considerations. Polym. Eng. Sci..

[35] Leung S.N., Park C.B., Xu D., Li H., Fenton R.G. (2006). Computer Simulation of Bubble-Growth Phenomena in Foaming. Ind. Eng. Chem. Res..

[36] Rahimidehgolan F., Altenhof W. (2023). Compressive behavior and deformation mechanisms of rigid polymeric foams: A review. Compos. Part B Eng..

[37] Soloveva O.V., Solovev S.A., Vankov Y.V., Shakurova R.Z. (2022). Experimental Studies of the Effective Thermal Conductivity of Polyurethane Foams with Different Morphologies. Processes.

[38] Weiser E.S., Johnson T.F., St Clair T.L., Echigo Y., Kaneshiro H., Grimsley B.W. (2000). Polyimide Foams for Aerospace Vehicles. High Perform. Polym..

[39] Ou A., Huang Z., Qin R., Chen X., Li Y., Liu Y., Liu X., Wang X. (2019). Preparation of Thermosetting/Thermoplastic Polyimide Foam with Pleated Cellular Structure via In Situ Simultaneous Orthogonal Polymerization. ACS Appl. Polym. Mater..

[40] Xu L., Jiang S., Li B., Hou W., Li G., Memon M.A., Huang Y., Geng J. (2015). Graphene Oxide: A Versatile Agent for Polyimide Foams with Improved Foaming Capability and Enhanced Flexibility. Chem. Mater..

[41] Yao Y., Zhang G., Li J., Wang A., Shi X. (2018). Effects of 4,4’-diaminodiphenyl ether on the structures and properties of isocyanate-based polyimide foams. J. Appl. Polym. Sci..

[42] Tian H., Yao Y., Ma S., Fu L., Xiang A., Rajulu A.V. (2017). Improved mechanical, thermal and flame resistant properties of flexible isocyanate-based polyimide foams by graphite incorporation. High Perform. Polym..

