# Supplementary material for: Preparation and Properties of High-Temperature-Resistant, Lightweight, Flexible Polyimide Foams with Different Diamine Structures
Source: Polymers (Basel). 2023 Jun 8;15(12):2609. doi: 10.3390/polym15122609 (PMC10304918; doi:10.3390/polym15122609)
Supplement: Supplementary file 1 [file polymers-15-02609-s001.zip › polymers-2398583-supplementary.pdf]

Supporting Information

Preparation and properties of high temperature resistant, lightweight flexible polyimide foams with different diamine structures

*Shuhuan Yun, Xianzhe Sheng, Shengli Wang, Xing Miao, Xuetao Shi, Yongsheng Zhao, Jianbin Qin, and Guangcheng Zhang**

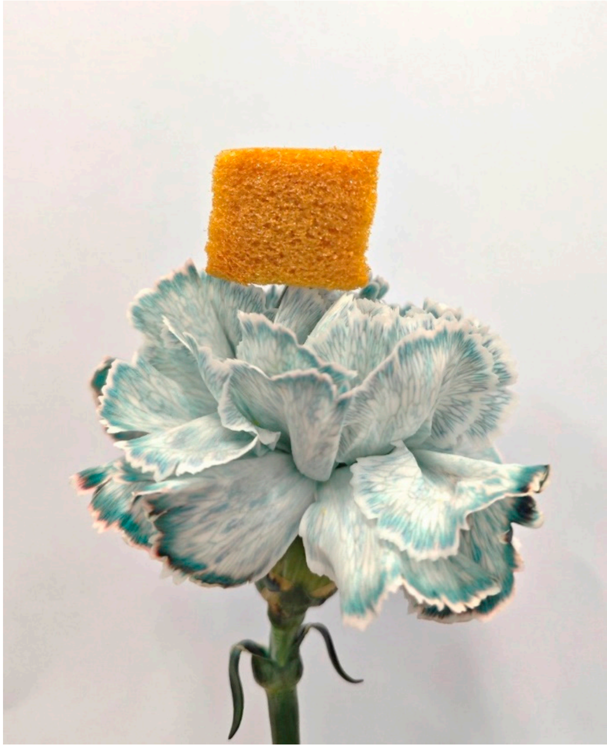

Figure S1. Appearance photos of $PIF_{BTDA-ODA}$

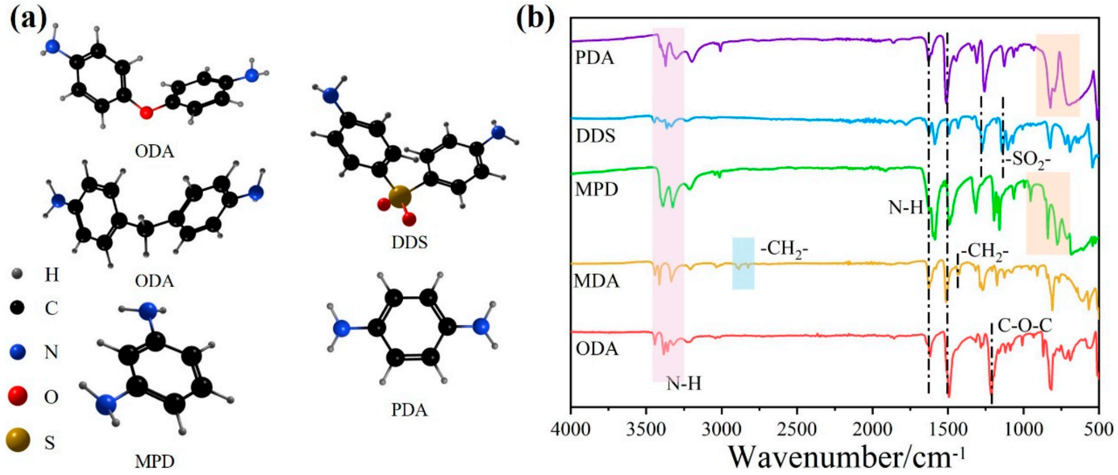

Figure S2. Diamine molecular structure models (a) and FTIR spectrum of diamine monomers (b).

Table S1. Mass changes before and after drying and residual solvent contents of PEAS precursors.

PEAS samples	Mass before drying/g	Mass after drying/g	Residual solvent content/%
	1.0455	0.9121	12.76
$PEAS_{BTDA\text{-}ODA}$	1.0037	0.8798	12.34
	1.2108	1.0551	12.86
	1.2381	1.0701	13.57
$PEAS_{BTDA\text{-}MDA}$	1.3455	1.1573	13.99
	0.9773	0.8465	13.38
	1.1178	0.9701	13.21
$PEAS_{BTDA\text{-}MPD}$	1.045	0.9020	13.68
	1.1636	1.0096	13.23
	1.0918	0.8961	17.92
$PEAS_{BTDA\text{-}DDS}$	1.0738	0.8860	17.49
	1.2536	1.0348	17.45
	1.1549	1.0080	12.72
$PEAS_{BTDA\text{-}PDA}$	1.2745	1.1097	12.93
	1.3417	1.1645	13.21

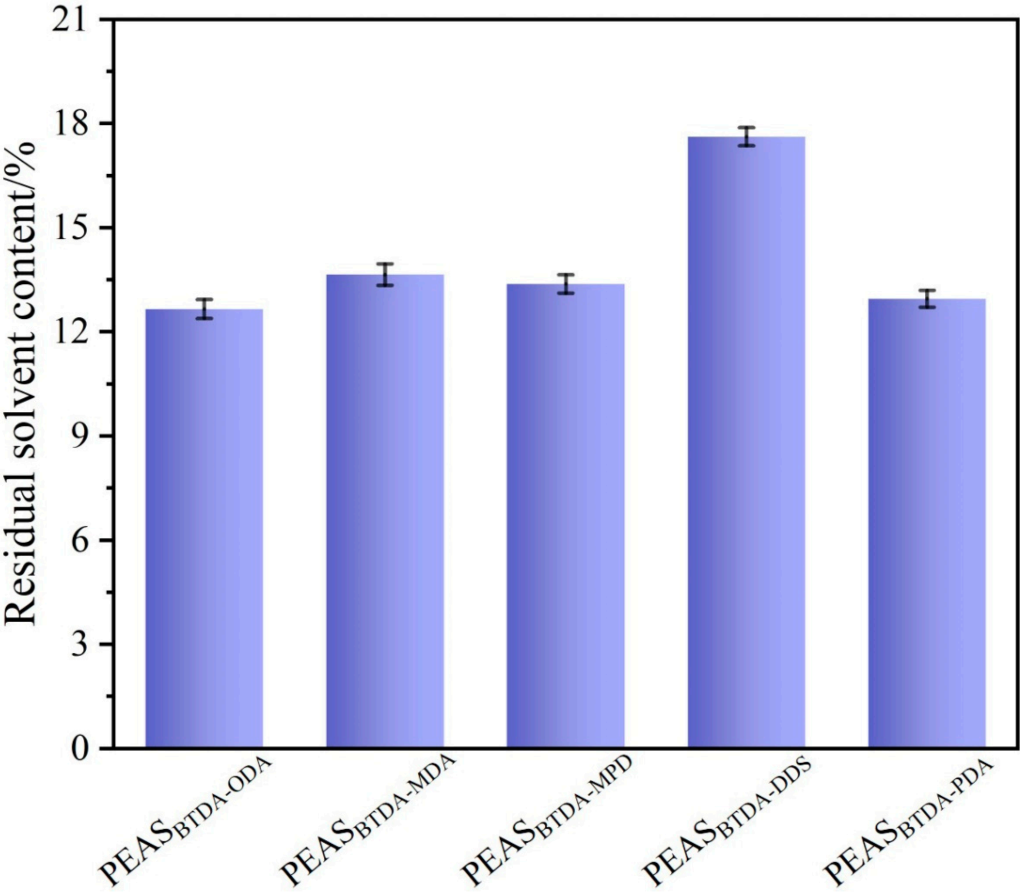

Figure S3. The residual solvent contents of PEAS precursors.

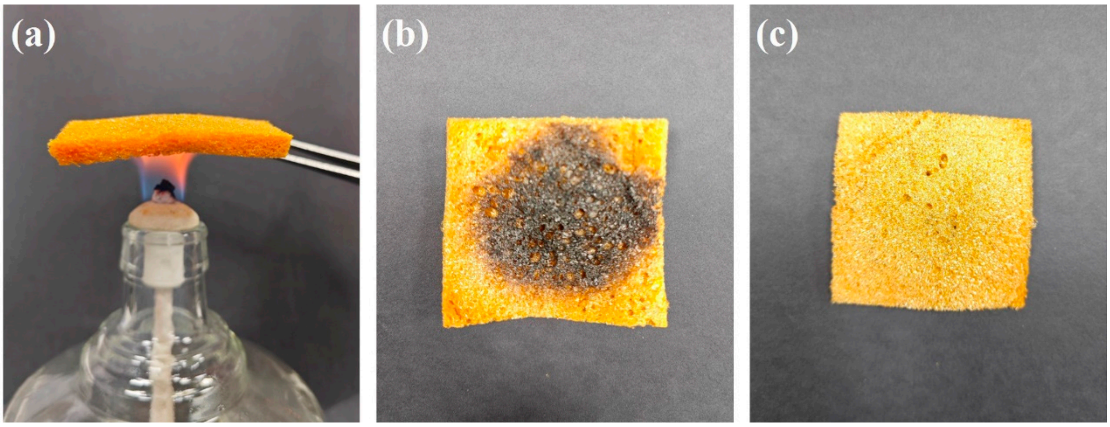

Figure S4. Flame retardant effect of PIF. Heating the foam with an alcohol lamp (a), the state of the heated surface after three minutes of heating (b), the state of the back of the foam after three minutes of heating (c).

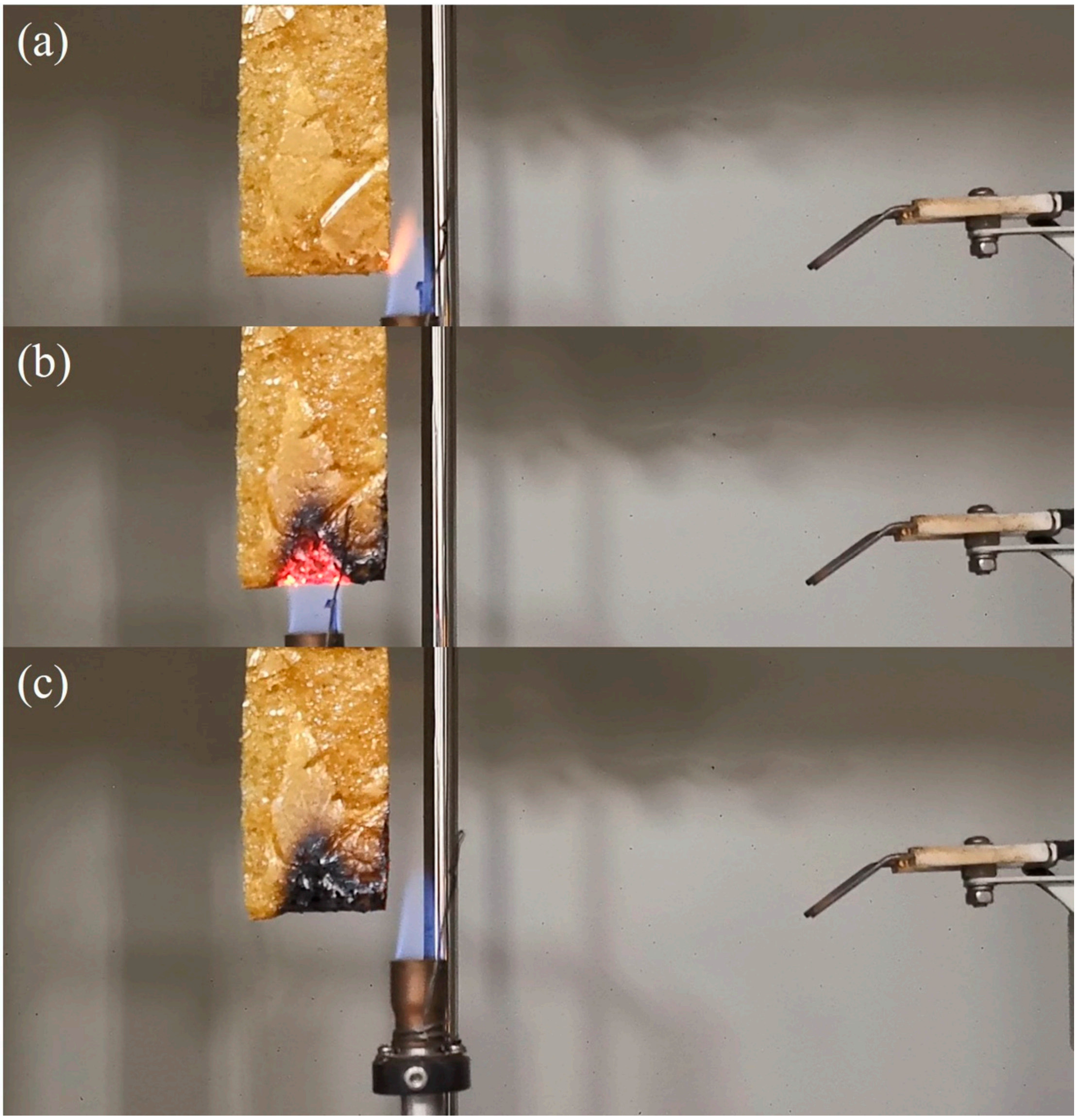

Figure S5. The state of the sample before vertical combustion (a), the state of the sample in vertical combustion (b), The state of the sample after 25 s vertical combustion (c).
